# Supplementary material for: Hybrid Models and Biological Model Reduction with PyDSTool
Source: PLoS Comput Biol. 2012 Aug 9;8(8):e1002628. doi: 10.1371/journal.pcbi.1002628 (PMC3415397; doi:10.1371/journal.pcbi.1002628)
Supplement: Text S4 — Complete source code for the PyDSTool package (version 0.88.120504). Includes API documentation and help files linking to web pages. This file is identical to the current public release on Sourceforge.net. (ZIP) [file pcbi.1002628.s004.zip › PyDSTool/html/PyDSTool.Toolbox.optimizers.helpers.finite_difference.ForwardFiniteDifferences-class.html]

xml version="1.0" encoding="ascii"?


PyDSTool.Toolbox.optimizers.helpers.finite\_difference.ForwardFiniteDifferences


| Home | Trees | Indices | Help | PyDSTool |
| --- | --- | --- | --- | --- |

Package PyDSTool ::
Package Toolbox ::
Package optimizers ::
Package helpers ::
Module finite\_difference ::
Class ForwardFiniteDifferences

[hide private]

[frames] | no frames]

# Class ForwardFiniteDifferences

source code

```
               object --+    
                        |    
FiniteDifferencesFunction --+
                            |
                           ForwardFiniteDifferences
```

---

A function that will be able to computes its derivatives with a
forward difference formula


|  |  |
| --- | --- |
| Instance Methods | [hide private] |

|  |  |
| --- | --- |
| \_\_call\_\_(self, params) | source code |

|  |  |
| --- | --- |
| \_\_init\_\_(self, eps=1e-07, \*args, \*\*kwargs)  Creates the function :... | source code |

|  |  |
| --- | --- |
| gradient(self, params)  Computes the gradient of the function | source code |

|  |  |
| --- | --- |
| hessian(self, params)  Computes the hessian of the function | source code |

|  |  |
| --- | --- |
| hessianvect(self, params)  Computes the hessian times a vector | source code |

**Inherited from `FiniteDifferencesFunction`**:
`residual`

**Inherited from `object`**:
`__delattr__`,
`__getattribute__`,
`__hash__`,
`__new__`,
`__reduce__`,
`__reduce_ex__`,
`__repr__`,
`__setattr__`,
`__str__`


|  |  |
| --- | --- |
| Properties | [hide private] |

**Inherited from `object`**:
`__class__`


|  |  |
| --- | --- |
| Method Details | [hide private] |

|  |  |
| --- | --- |
| \_\_init\_\_(self, eps=1e-07, \*args, \*\*kwargs)  *(Constructor)* | source code |

```
Creates the function :
- eps is the amount of difference that will be used in the computations
```

Overrides: object.\_\_init\_\_

  


| Home | Trees | Indices | Help | PyDSTool |
| --- | --- | --- | --- | --- |

|  |  |
| --- | --- |
| Generated by Epydoc 3.0.1 on Fri May 4 15:24:09 2012 | http://epydoc.sourceforge.net |
